# Foot progression angle estimation using a single foot-worn inertial sensor

**DOI:** 10.1186/s12984-021-00816-4

**Published:** 2021-02-17

**Authors:** Frank J. Wouda, Stephan L. J. O. Jaspar, Jaap Harlaar, Bert-Jan F. van Beijnum, Peter H. Veltink

**Affiliations:** 1 Faculty of Electrical Engineering, Mathematics and Computer Science, University of Twente, Enschede, The Netherlands; 2 Department of Rehabilitation Medicine, Amsterdam Movement Sciences, VU University Medical Center, Amsterdam, The Netherlands; 3 Department of Biomechanical Engineering, Delft University of Technology, Delft, The Netherlands

**Keywords:** Foot progression angle, Inertial sensors, Knee osteoarthritis, Minimal sensing, Zero Velocity Update, PCA

## Abstract

**Background:**

The foot progression angle is an important measure used to help patients reduce their knee adduction moment. Current measurement systems are either lab-bounded or do not function in all environments (e.g., magnetically distorted). This work proposes a novel approach to estimate foot progression angle using a single foot-worn inertial sensor (accelerometer and gyroscope).

**Methods:**

The approach uses a dynamic step frame that is recalculated for the stance phase of each step to calculate the foot trajectory relative to that frame, to minimize effects of drift and to eliminate the need for a magnetometer. The foot progression angle (FPA) is then calculated as the angle between walking direction and the dynamic step frame. This approach was validated by gait measurements with five subjects walking with three gait types (normal, toe-in and toe-out).

**Results:**

The FPA was estimated with a maximum mean error of ~ 2.6° over all gait conditions. Additionally, the proposed inertial approach can significantly differentiate between the three different gait types.

**Conclusion:**

The proposed approach can effectively estimate differences in FPA without requiring a heading reference (magnetometer). This work enables feedback applications on FPA for patients with gait disorders that function in any environment, i.e. outside of a gait lab or in magnetically distorted environments.

## Background

Knee osteoarthritis (KOA) is among the most reported musculoskeletal diseases (men 10.1%, women 13.6%) and the leading cause for disability among the elderly [1, 2]. This disease has no cure currently, however, patients can make use of surgical, pharmacological and biomechanical treatments to improve their quality of life [3]. Pharmacological treatment can reduce the effects of symptoms of KOA, in severe stages of the disease surgical treatment (knee replacement) could be considered [4]. Biomechanical treatment can help to reduce the knee loading, which has been shown to correlate with pain, cartilage degeneration and disease progression [5].

Biomechanical treatment can be achieved by use of braces, canes and/or gait retraining. No additional devices are required for gait retraining, however as this treatment is time consuming and space-bounded it has not been adopted on a large scale [6]. The goal of gait retraining is to reduce the loading on the knee by gradually modifying the patients’ gait pattern [6]. Directly measuring the medial knee loading would require invasive force sensors and is therefore only possible after a knee replacement [7]. Alternatively, the medial knee loading can be estimated using a surrogate measure, namely the knee adduction moment (KAM) [8]. The KAM can be estimated using inverse dynamics, which requires a full-body motion capture system and force measurements [9].

However, the KAM is not an optimal parameter to provide feedback to patients, since the relation to kinematic parameters is not evident to them [10]. Therefore, instructing patients using a kinematic adaptation (toe-in gait) results in more effective decrease of the KAM [11–13]. This can be quantified using the foot progression angle (FPA), which is defined as the angle between the heading direction and foot orientation.

A recent study has shown that the FPA can effectively be measured using one foot-worn sensor [14], consisting of an accelerometer, gyroscope and magnetometer. However, the use of a magnetometer limits applications of this approach, since it requires a minimally disturbed Earth magnetic field. In various environments this is not the case, due to ferro-magnetic materials present in floors and walls [15]. With inaccurate measurements of the Earth magnetic field, no proper reference frame can be determined (errors as large as 20° in the heading direction have been observed near floors [15]), hence inaccurate estimates of the FPA are obtained.

To the best of our knowledge, there is no single-sensor approach for estimating FPA in any environment (including magnetically disturbances). This resulted in the following aim of this study: design and evaluation of an approach to estimate FPA using a single foot-worn inertial sensor (accelerometer and gyroscope). Due to using a dynamic foot reference frame instead of an Earth reference frame no magnetometer is required. To minimize the effects of drift during a single step, the Zero Velocity Update (ZUPT) is applied [16]. The accuracy of the proposed method is validated using an optical reference system. The findings of this study could have potential for future applications in feedback systems for KOA patients.

## Methods

This section describes the proposed method and the measurement protocol.

### FPA estimation

**Fig. 1:**
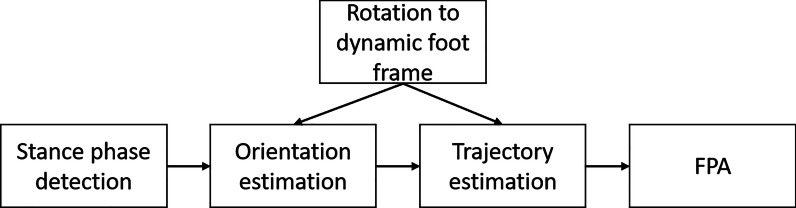
Flowchart of the proposed FPA estimation algorithm. Steps in the proposed FPA estimation algorithm are as follows: detect the stance phase, initiate the dynamic foot frame, estimate orientation of the foot, estimate the foot trajectory, and use this information to estimate the FPA

**Fig. 2:**
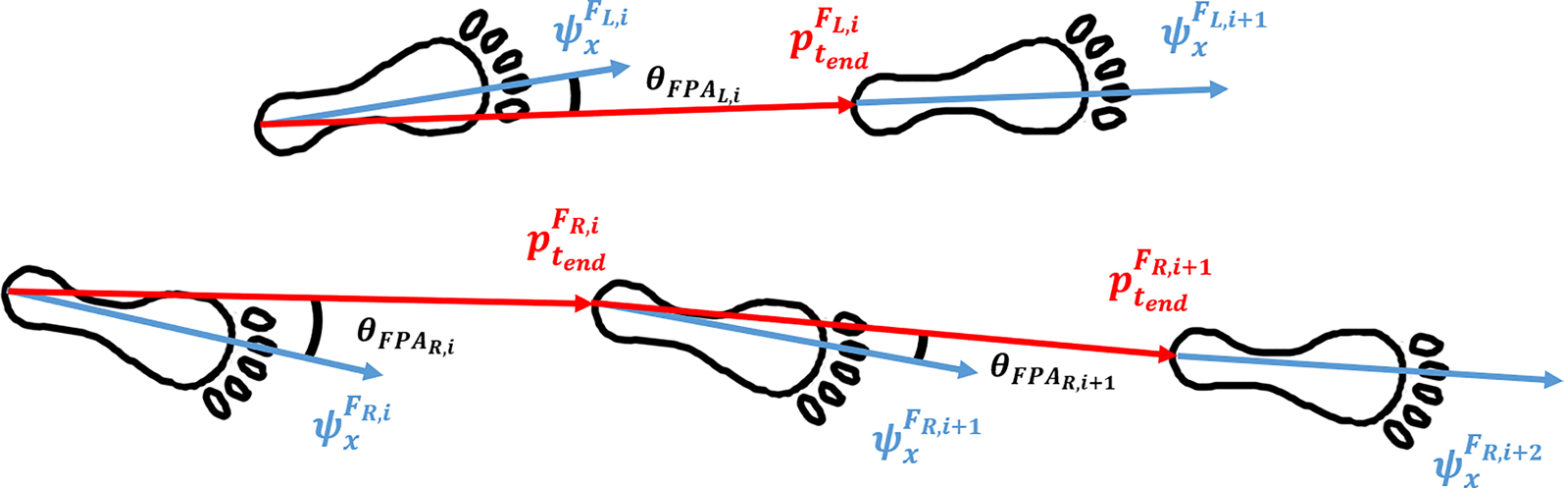
FPA definition. A dynamic foot frame, that is initialized in every stance phase *i* (for left (L) and right (R) separately) and is maintained until the consecutive stance phase of the same foot, is used for calculating the FPA as the angle between the foot direction and the walking direction. The x-direction of this dynamic foot frame ($\psi _x^{F_{L/R,i}}$) aligns with the foot direction. All signals are integrated in this dynamic foot frame to obtain a foot trajectory that ends at the next stance phase. This walking direction is shown in red and defined as the position vector between the calcaneus of two consecutive stance phases (with $p^{F_{L/R,i}}_{t_{end}}$, where $t_{end}$ is the step duration). This allows for direct computation of the FPA

Our proposed FPA estimation approach consists of five steps as schematically displayed in Fig. 1. The approach uses a dynamic foot frame (as schematically displayed in Fig. 2), which changes from stance phase *i* to next stance phase $i+1$. This is done by integration of angular velocity during a step in between subsequent stance phases [17], updated with Zero Angular Velocity Update (ZAVU). Therefore, the start and end of a step should be determined using a zero-velocity detection [18, 19]. With strap-down integration, the vector from calcaneus position in stance phase *i* to calcaneus position in stance phase $i+1$ is determined in this dynamic foot frame. Subsequently correcting for drift using ZUPT and zero vertical position at the start and end of the step. FPA is calculated from the angle between foot direction during stance phase *i* and direction of the next step [12], which is estimated based on the endpoint of the trajectory estimation, as schematically displayed in Fig. 2.

#### Stance phase detection

During the stance phase there are moments that the foot is approximately still on the ground, hence these moments can be identified using a zero-velocity detection approach [19]. Jimenez et al. developed three conditions for the detection approach, however, this resulted in some cases of short stance phases. Therefore, a fourth condition was included that ensures a minimal length of the stance phase. The following four conditions were used in the current study:

1. Norm of the acceleration vector needs to be between 9.0 and 11.0 $\frac{m}{s^2}$ (at time *t*).
2. The local variance ($\sigma ^2$) of the norm of the acceleration vector should be smaller than 0.5 $\frac{m^2}{s^4}$ (averaged over $2s + 1$ samples, which resulted in a time period of 0.11 s, with an experimentally determined $s = 5$ samples) during the stance phase to fulfill this condition, and is defined as:
  1 $$\begin{aligned} \sigma ^2 = \frac{1}{2s+1} \sum _{j = t - s}^{t + s}(a_{j} -\bar{a}_{t})^2 \end{aligned}$$
  where the local mean (of the norm of the acceleration vector) is defined as:
  2 $$\begin{aligned} \bar{a}_{t} = \frac{1}{2s+1} \sum _{j = t - s}^{t + s}a_{j} \end{aligned}$$
3. Norm of the angular velocity vector should be smaller than 50 $\frac{^{\circ }}{s}$ (at time *t*).
4. Stance phase length should be 16 ms at minimum, which ensures that the detection method does not suffer from potential false zero-velocity detections.

#### Mapping foot frame in sensor frame

**Fig. 3:**
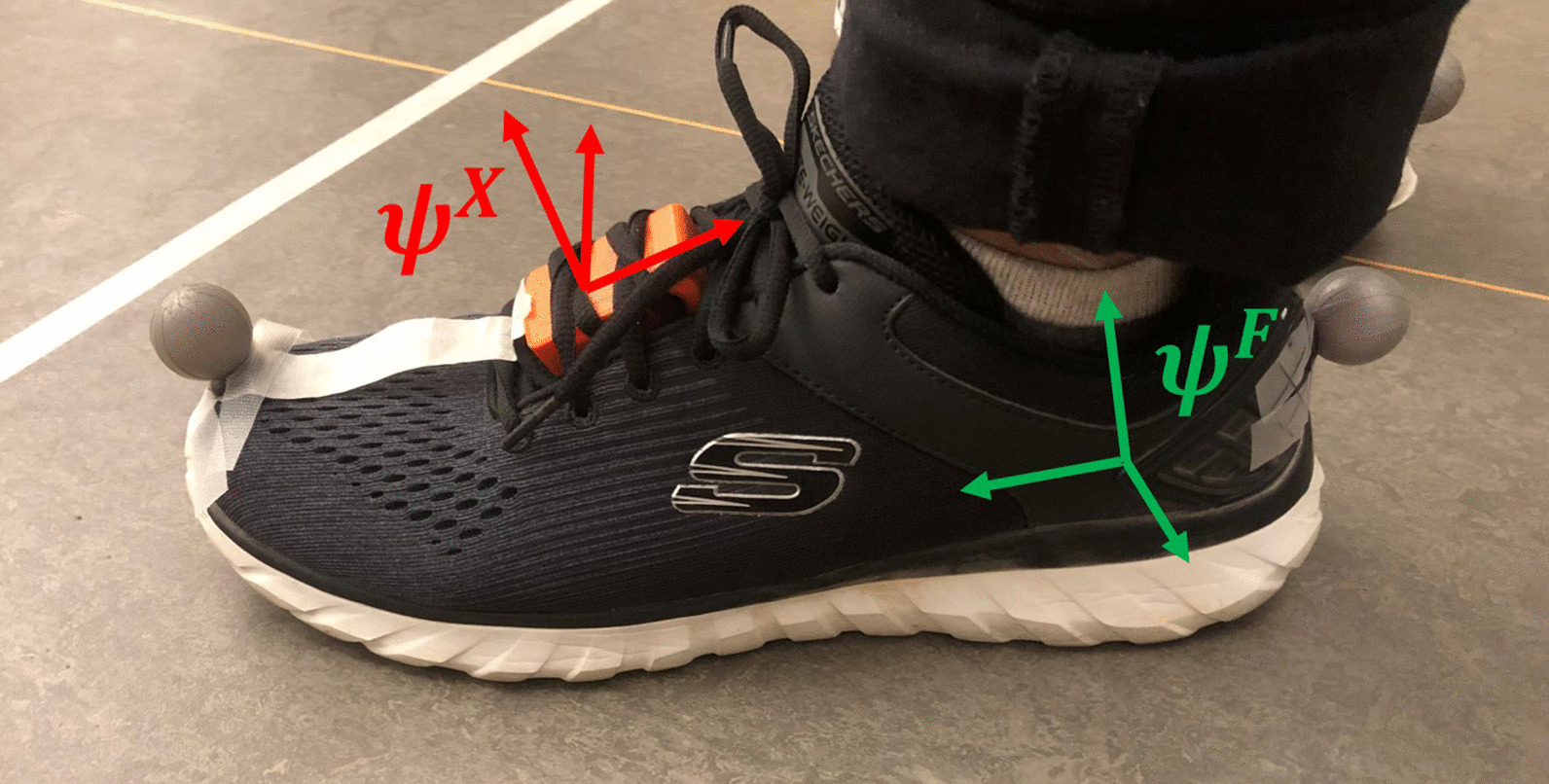
Measurement setup. An IMU is secured under the shoelaces and its’ coordinate system ($\psi ^X$) is displayed in red. The retroflective markers are places on the second metatarsal and the calcaneus, which is assumed to be a FPA of 0°. The foot reference coordinate system is shown in green ($\psi ^F$)

A mapping between the sensor frame ($\psi ^X$, red in Fig. 3) and the foot reference frame ($\psi ^F$, green in Fig. 3) is required to perform all calculations in $\psi ^F$. This is a fixed rotation, assumed that the sensor does not move relative to the foot. Subjects should perform the following calibration: stand still for 5 s, walk four steps with a FPA of $0^{\circ }$ (i.e., keep foot orientation as straight as possible). The first part of the calibration is used to determine the vertical axis ($f_z$) of $\psi ^F$ using the measured gravitational acceleration. The axis perpendicular to the foot direction ($f_y$) is determined in the dynamic part of the calibration. Principal Component Analysis (PCA) of the angular velocity is used to determine a common rotation axis ($f_y$) [20]. The third axis is determined by the cross-product of the other two axes (as it should be perpendicular to both previously defined axes):

3 $$\begin{aligned} f_x = f_y \times f_z \end{aligned}$$

This determines the axis in direction of the foot, i.e., this definition allows for FPA calculation using the angle between heading direction of the step and foot direction axis. To ensure a proper coordinate system ($f_y$ perpendicular to $f_z$ and thus in the horizontal plane), $f_y$ was subsequently determined by taking the cross-product of $f_x$ and $f_z$. The mapping of $\psi ^X$ to $\psi ^F$ can then by performed using the following (constant) rotation matrix ($R^{XF}$):

4 $$\begin{aligned} R^{XF} = \begin{bmatrix} f_x&f_y&f_z \end{bmatrix}^T \end{aligned}$$

#### Orientation estimation

**Fig. 4:**
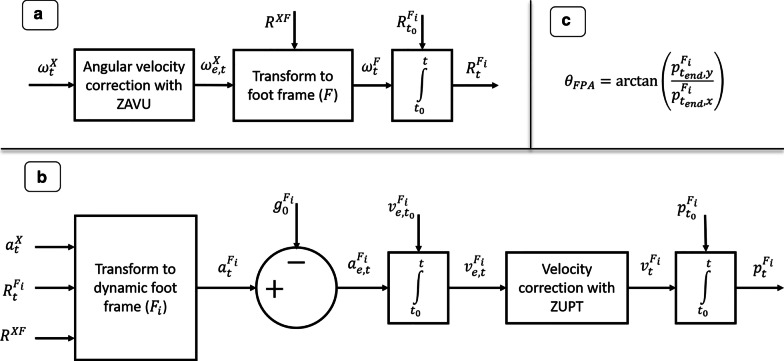
Steps in the FPA estimation approach. FPA estimation approach using gyroscope ($\omega _t^X$) and accelerometer ($a_t^X$) data: **a** sensor angular velocity is corrected using the Zero Angular Velocity Update (ZAVU). Next, it is rotated to the foot frame ($\psi ^{F}$) by using the mapping between the sensor and foot frames ($R^{XF}$). The orientation of the dynamic foot frame ($R^{F_i}_t$) is determined by integrating this angular velocity and initializing it with $R^{F_i}_{t_0} = I$. **b** Acceleration information is rotated to the dynamic foot frame ($\psi ^{F_i}$), such that the gravitational acceleration can be subtracted to obtain the estimated free acceleration ($a^{F_i}_{e,t}$). This is integrated to velocity ($v^{F_i}_{e,t}$) by initializing it with $v^{F_i}_{t_0} = 0$, which in turn is corrected using ZUPT. After another integration step (initialized with $p^{F_i}_{t_0} = 0$) the position of the foot is calculated in the dynamic foot frame. **c** Since everything is calculated in $\psi ^{F_i}$ the FPA is estimated using a trigonometric relation of the foot position at the end of the step ($t_{end}$)

Start of a step is defined as the middle of a determined zero-velocity phase (according to the mentioned 4 conditions). The angular velocity is measured in $\psi ^X$, which is rotated to $\psi ^F$ by using the determined sensor to foot frame mapping $R^{XF}$ (Fig. 4a). The dynamic foot reference frame at step *i* ($\psi ^{F_i}$) is initialized by an identity matrix ($R^{F_i}_{t_0}$), such that the change with respect to this frame can be evaluated using the following differential equation [21]:

5 $$\begin{aligned} \dot{R}_t^F = \tilde{\omega }^F R_t^F \end{aligned}$$

with $\tilde{\omega }$ as the skew matrix of the angular velocity, which is defined as:

6 $$\begin{aligned} \tilde{\omega }^F = \begin{bmatrix} 0 &{} -\omega _z^F &{} \omega _y^F \\ \omega _z^F &{} 0 &{} -\omega _x^F \\ -\omega _y^F &{} \omega _x^F &{} 0 \end{bmatrix} \end{aligned}$$

#### Trajectory estimation

**Fig. 5:**
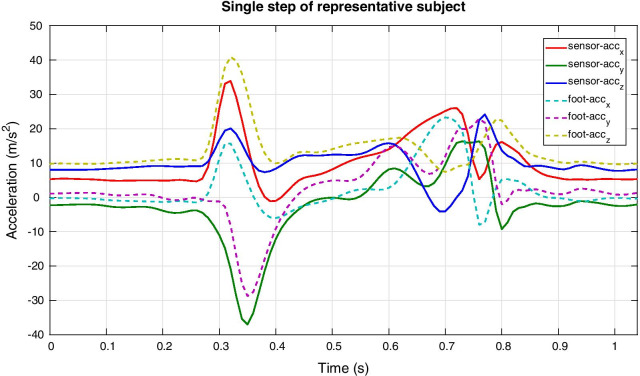
Acceleration profiles of representative subject. The acceleration ($A^X_t$) profiles (shown in solid lines) before transforming them to the dynamic foot frame as shown in Fig. 4 are compared to the accelerations ($A^{F_i}_t$) when this transformation has been applied (shown in dashed lines)

Figure 4b shows the different steps to obtain the foot position ($p_t^{F_i}$). First the measured acceleration ($a_t^X$) should be transformed to $\psi ^{F_i}$ at any time *t* during step *i*, such that the gravity component can be subtracted, as can be seen from an example step of a representative subject provided in Fig. 5. After this step the acceleration is integrated to obtain the velocity ($v_t^F$). Since it is known that the velocity should be zero at the next stance phase, we can apply a linear correction (to account for the potential drift) to the velocity vector from start to end of the step. A second integration step is applied to obtain the foot position w.r.t. start of the step ($p_t^F$).

#### FPA

The FPA is estimated using the heading vector (endpoint $p^{F_i}_{t_{end}}$ of step *i*) which is expressed in $\psi ^{F_i}$, therefore the following direct trigonometric relation is applicable here:

7 $$\begin{aligned} \theta _{FPA} = \arctan {\frac{p^{F_i}_{t_{end},y}}{p^{F_i}_{t_{end},x}}} \end{aligned}$$

### Validation measurement protocol

The accuracy of the proposed FPA estimation approach is quantified by comparing results obtained with our approach to those from using an optical motion capture system. Five healthy volunteers (5 males; age: $25.2\pm 4.2$ years; height: $1.83\pm 0.09$ m; weight: $80.0\pm 9.5$ kg; body mass index: $24.1\pm 3.4$ kg/m$^2$) participated in this research in a gait laboratory. All subjects reported no recent injuries that affect balance or mobility. The ethics committee of the Faculty of Electrical Engineering, Mathematics and Computer Science at the University of Twente approved this protocol and all subjects provided written informed consent prior to the measurements.

Subjects are fitted with one inertial sensor (MTw Awinda, Xsens, Enschede, the Netherlands) on top of the shoe of both feet and two retro-reflective markers (placed on the head of the second metatarsal and the calcaneus, as shown in Fig. 3). The MTw is a wireless inertial sensor that transmits data (at 100 Hz) over Bluetooth, which is recorded using MT Software Suite (Xsens, Enschede, the Netherlands). The position of retro-reflective markers is recorded (at 100 Hz) using eight high-speed infrared cameras (Vicon, Oxford, UK) and processed with Nexus 2.8.2 (Vicon, Oxford, UK). To compare the FPA outcomes of both systems, a synchronization between the inertial and optical systems is required. This is achieved by stamping the right foot at the ground for the start of the measurement. This signal is present in both the optical and inertial measurement data and is used to align both signals. Small misalignments (1–5 ms) are allowable since we are interested in the FPA per different step and not at discrete time indexes.

The reference FPA was determined based on the markers placed on the calcaneus and second metatarsal by calculating the angle between the line connecting both retro-reflective markers and the walking direction vector (defined by a line between calcaneus of the same foot in different stance phases) in the lab reference frame (depends on the camera calibration) [13].

Every measurement started with a 0° FPA calibration (for the inertial approach), which consists of a static part and a dynamic part (as mentioned in Section: Mapping foot frame in sensor frame). Subjects should remain as still as possible with feet pointing forwards (0° FPA) for approximately five seconds, to determine the gravitational axis. Subsequently, subjects were asked to walk with a zero degrees FPA for four steps. A visual reference is provided to subjects by a tape placed on their shoe (shown in Fig. 3), which shows the foot direction vector. By aligning this with a line on the floor subjects could achieve a FPA close to zero, which was evaluated using the optical reference.

After this calibration trial, subjects were asked to perform three sets of 12 trials of walking in a straight line within the measurement volume of the optical motion capture system (10 × 4 m, projected on the floor). Each set of 12 trials consists of walking at their preferred walking speed with either normal, positive (toe-out) or negative (toe-in) FPA. The difference in FPA between each of these three walking conditions was self-selected by the subjects, to let the FPA variations be within the range of acceptable angles.

A difference between FPA estimates for the optical and inertial approach was used for an evaluation of the accuracy of the proposed inertial FPA estimation approach. We decided not to evaluate a root mean squared difference but a mean difference, because the sign of errors is relevant in this situation due to potential spatial misalignment of the 0° FPA. After correction for the determined offset, results are presented using a Bland–Altman plot to show the distribution of FPA measured by both the optical and inertial sensing approach [22]. Additionally, a repeated measures one-way ANOVA test [23] is performed to determine if both the inertial and optical approaches can differentiate between the three gait conditions.

## Results

**Fig. 6:**
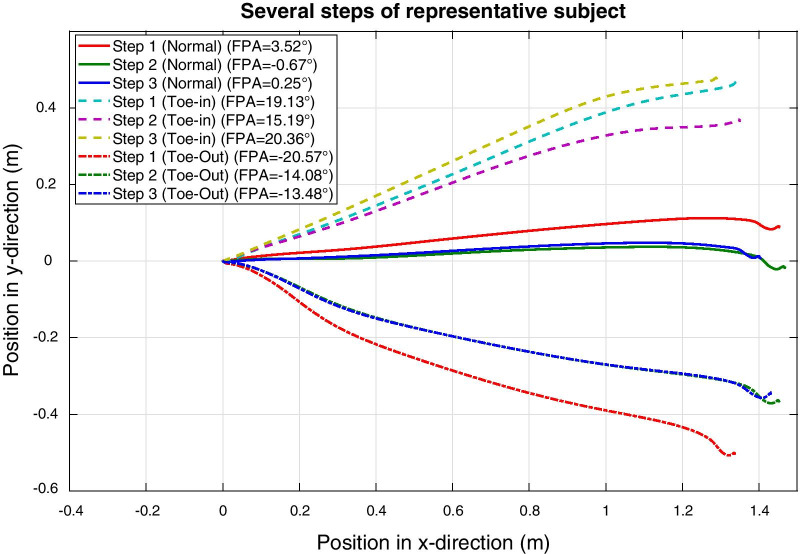
Two-dimensional foot trajectories. Trajectories of the foot of one representative subject in the horizontal plane of the dynamic foot frame during three consecutive steps for each FPA condition (normal, toe-in and toe-out). All steps start in the origin since the dynamic foot frame is defined to start at zero. The final position of the foot during a step is used to determine the FPA according to Eq. (7) and is shown in the legend for each step

Figure 6 shows the foot trajectories within the dynamic foot frame in the horizontal plane of a representative subject, which are all originating from the origin and the end position is used for determining the FPA according to Eq. (7).

Table 1 shows the mean differences (and standard deviation) between the FPA estimated using retro-reflective markers and using the proposed inertial approach. It can be seen that subjects (e.g., S01 and S05) with larger differences (up to 5°) compared to the optical reference also have larger 0° FPA calibration differences.

**Fig. 7:**
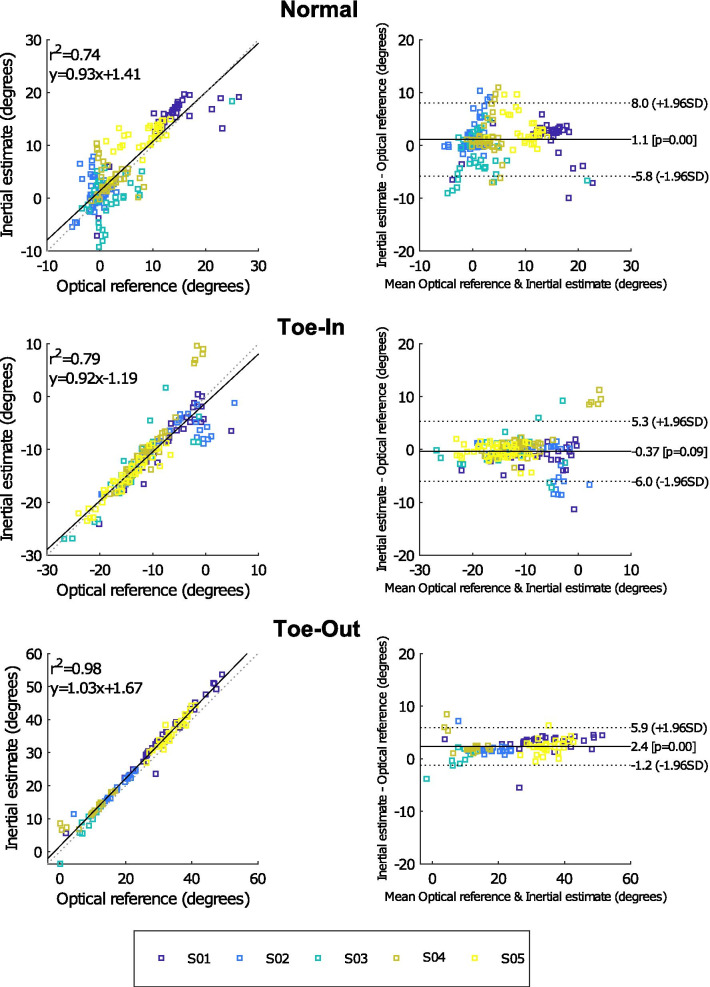
Bland–Altman comparison. Bland–Altman graphs comparing the FPA estimated with an optical and inertial approach for five subjects. The mean observed differences during the 0° FPA calibration trial was added to the inertial outcomes of the individual subjects, such that impact of misalignment of the 0° FPA axes is minimal. Different graphs are presented for the three types of gait (normal, toe-in and toe-out), for each condition approximately 40 steps were analyzed. Please note the differences in angle ranges between the three types of gait

**Table 1:** The mean (and standard deviation) FPA differences (in degrees) of the optical approach compared to the inertial approach

Subjects	Gait			0° FPA
	Normal	Toe-in	Toe-out	Calibration
S01	− 5.22 (± 3.35)	− 2.39 (± 2.61)	− 6.58 (± 1.72)	− 3.99 (± 0.37)
S02	0.01 (± 2.83)	2.51 (± 2.86)	− 0.21 (± 1.41)	1.38 (± 0.75)
S03	1.85 (± 4.04)	0.10 (± 3.43)	− 0.13 (± 1.87)	− 0.17 (± 2.82)
S04	0.20 (± 3.43)	0.31 (± 3.25)	− 1.45 (± 2.05)	1.68 (± 2.86)
S05	1.69 (± 2.67)	5.15 (± 1.41)	2.36 (± 1.36)	4.71 (± 1.14)

A more detailed comparison between the FPA (of individual steps) estimated from an optical and inertial approach can be found in Fig. 7, after correcting for the 0° FPA calibration differences. The correlation between both approaches is shown by the plots on the left (for all three types of gait). Good correlation coefficients ($r^2 > 0.7$) can be observed for all conditions. Furthermore, the mean bias between the inertial approach and the optical reference is small ($< 2.5^{\circ }$) for all conditions.

Results of a repeated measures one-way ANOVA test between the different gait conditions show that both the inertial approach and the optical reference system can significantly ($p<< 0.01$) discriminate between those conditions. These results were obtained for all subjects and both measurement approaches.

## Discussion

The aim of this research was to evaluate an approach for estimating FPA using a single foot-worn inertial (accelerometer and gyroscope) sensor. The proposed approach uses a dynamic step reference frame to calculate the FPA of each step with respect to the foot frame during stance. A comparison with an optical reference shows good correlation and it can effectively differentiate between the different types of gait (normal, toe-in and toe-out).

Table 1 shows that an offset between the optical and inertial approach could have impacted the observed differences between both approaches. Such an offset is expected to be the result of a misalignment between the defined 0° FPA for both approaches. To that end, results presented in Fig. 7 were corrected for the observed differences during the 0° FPA calibration measurement (by adding the mean observed offset in Table 1 to the estimated FPA with the inertial approach for each subject individually). This misalignment can occur during the sensor to foot calibration of the inertial approach, since subjects were instructed to walk with an FPA of 0° using optical feedback (tape on the shoe and lines on the floor). Furthermore, misplacement of the retro-reflective markers could also result in an offset between both approaches. The inertial calibration procedure could be improved by using a board with cut-outs for the feet, which forces subjects to walk with 0° FPA. In this manner, a potential misplacement of the retro-reflective markers can also be determined. However, it should be noted that differences in inertial sensor placement have less impact on the estimation accuracy than the execution of the calibration procedure.

Related works of estimating foot angles using inertial sensing reported comparable range of FPAs, resulting in similar performance as our proposed approach (maximum mean difference of ~ 2.6°). Bidabadi et al. used a single foot-worn IMU to estimate the foot pitch angle (ankle flexion/extension) and reported a mean accuracy of ~ 3.8° [24]. Huang et al. presented a single foot-worn IMU (with magnetometer) approach for estimating the FPA with a maximum mean error of ~ 2.5° [14]. While a full-body inertial approach for estimating FPA was reported to have an error of ~ 2.4° [4]. However, these approaches require more on-body sensors or cannot be used in all (magnetically distorted) measurement environments.

One of the issues with inertial sensing is that directly integrating the accelerometer and gyroscope measurements will result in drift of the sensor position/orientation. However, impact of such drift increases over time, i.e., short-term integration could result in outcomes with acceptable accuracy. To that end, we applied two ways of minimizing such effects, namely ZUPT and integrating over each individual step separately. ZUPT allowed for linear corrections to the obtained velocity/position, due to the known zero-velocity state during stance. And the use a dynamic step frame allows for integration of accelerometer data during each individual step. In this manner, drift only impacts the estimated FPA during a single step, which reduces the negative effect on accuracy substantially.

The proposed approach has potential for real-time feedback applications, such as proposed by Karatsidis et al. [4]. A reduction in the number of sensors is beneficial to patients, because of the decreased complexity and costs. However, this approach was evaluated with healthy participants with no reported balance or mobility issues. The FPA of people with movement disorders might be estimated with lower accuracy using the proposed approach. Additionally, with different gait dynamics, the zero-velocity detection conditions might change. When the proposed conditions do not lead to adequately detected zero-velocity moments in a patient population, an alternative method could be to use gait event detection methods that have been evaluated for slow/impaired gait [25, 26]. Furthermore, the calibration procedure (walking with 0° FPA) used in this work might be difficult for people with a movement disorder. An alternative could be to perform repeated dorsal/plantar ankle flexions. However, in initial measurements this resulted in a rotation axis that was not perpendicular to the vertical since the rotation was not consistently in the horizontal plane. If the mapping of sensor to foot frame is known, the calibration procedure might be removed, e.g., in case of a shoe with an embedded IMU [27, 28] (this would also minimize artefacts caused by relative change in orientation between sensor and foot segment), and which will not suffer from magnetic disturbances with the proposed approach. Depending on the application the impact of an incorrect 0° FPA might vary, as long as differences compared to a baseline measurement can be measured with sufficient accuracy [29]. Another limitation of this work is that the FPA was evaluated for walking in a straight line, the impact of turns on the estimation accuracy would require additional research. In a future study, we propose to perform a sensitivity analysis to evaluate the influence of issues, like fixation of the sensor to the shoe and inaccuracies in the functional calibration protocol, on the performance of the proposed FPA algorithm in more detail. Specifically, with knee osteoarthritis patients to gain insight in the clinical applicability of this algorithm.

To apply the proposed approach in a (semi-)real-time feedback application a firmware implementation would be required. In the current study, the algorithm was off-line applied in MATLAB, however, minimal calculation time (~ 4 ms per step) was observed for this implementation. Furthermore, feedback can only be provided after the step is finished (due to uncertain step direction during swing phase). Therefore, it is expected that this method can provide (semi-)real-time feedback on the FPA. However, additional research is required to investigate the accuracy of the proposed approach in real-time and with patients.

## Conclusion

This work presented a novel approach to estimate FPA using information from a single foot-worn inertial sensor (accelerometer and gyroscope) that can be used in any (magnetically distorted) environment. Experimental results show that the proposed approach has good correlation with an optical reference system. Furthermore, differences between various types of gait (normal, toe-in and toe-out) can be discriminated with our approach. Therefore, this research could provide a basis for future research into the use of wearable feedback systems for gait training of KOA patients in any environment. Such research is required to determine if the proposed method is sufficient for reducing knee loading in KOA patients.

## Data Availability

The datasets used and/or analysed during the current study are available from the corresponding author on reasonable request.

## Abbreviations

FPA: Foot progression angle
KAM: Knee adduction moment
KOA: Knee osteoarthritis
ZAVU: Zero Angular Velocity Update
ZUPT: Zero Velocity Update
